# Selective production of the itaconic acid-derived compounds 2-hydroxyparaconic and itatartaric acid

**DOI:** 10.1016/j.mec.2024.e00252

**Published:** 2024-11-16

**Authors:** Philipp Ernst, Felicia Zlati, Larissa Kever, Astrid Wirtz, Rainer Goldbaum, Jörg Pietruszka, Benedikt Wynands, Julia Frunzke, Nick Wierckx

**Affiliations:** a Institute of Bio- and Geosciences IBG-1: Biotechnology, Forschungszentrum Jülich GmbH, Wilhelm-Johnen-Straße, 52428 Jülich, Germany; b Institute of Bioorganic Chemistry, Heinrich-Heine University Düsseldorf in Forschungszentrum Jülich GmbH, Wilhelm-Johnen-Straße, 52428, Jülich, Germany

**Keywords:** *Ustilago cynodontis*, 2-Hydroxyparaconic and itatartaric acid, Derivatives specificity, Metabolic engineering, Fermentation, Downstream processing

## Abstract

There is a strong interest in itaconic acid in the medical and pharmaceutical sectors, both as an anti-bacterial compound and as an immunoregulator in mammalian macrophages. Fungal hosts also produce itaconic acid, and in addition they can produce two derivatives 2-hydroxyparaconic and itatartaric acid. Not much is known about these two derivatives, while their structural analogy to itaconate could open up several applications. In this study, we report the production of these two itaconate-derived compounds. By overexpressing the itaconate P450 monooxygenase Cyp3 in a previously engineered itaconate-overproducing *Ustilago cynodontis* strain, itaconate was converted to its lactone 2-hydroxyparaconate. The second product itatartarate is most likely the result of the subsequent lactone hydrolysis. A major challenge in the production of 2-hydroxyparaconate and itatartarate is their co-production with itaconate, leading to difficulties in their purification. Achieving high derivatives specificity was therefore the paramount objective. Different strategies were evaluated including process parameters such as substrate and pH, as well as strain engineering focusing on Cyp3 expression and product export. 2-hydroxyparaconate and itatartarate were successfully produced from glucose and glycerol, with the latter resulting in a higher derivatives specificity due to an overall slower metabolism on this non-preferred carbon source. The derivatives specificity could be further increased by metabolic engineering approaches including the exchange of the native itaconate transporter Itp1 with the *Aspergillus terreus* itaconate transporter MfsA. Both 2-hydroxyparaconate and itatartarate were recovered from fermentation supernatants following a pre-existing protocol. 2-hydroxyparaconate was recovered first through a process of evaporation, lactonization, and extraction with ethyl acetate. Subsequently, itatartarate could be obtained in the form of its sodium salt by saponification of the purified 2-hydroxyparaconate. Finally, several analytical methods were used to characterize the resulting products and their structures were confirmed by nuclear magnetic resonance spectroscopy. This work provides a promising foundation for obtaining 2-hydroxyparaconate and itatartarate in high purity and quantity. This will allow to unravel the full spectrum of potential applications of these novel compounds.

## Highlights

• Engineered *Ustilago* for 2-hydroxyparaconate and itatartarate production.
• Cyp3 itaconate P450 monooxygenase is limiting production.
• Combination of strain- and process engineering enabled 100% specificity.
• Purified itaconate derivatives to enable application testing.

## Graphical abstract

**Figure:**
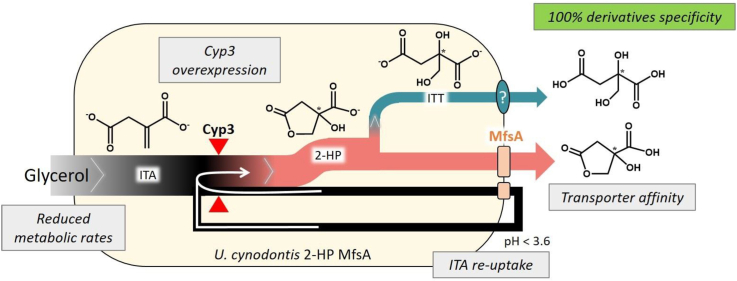

## 1 Introduction

*Ustilaginaceae* are dimorphic basidiomycetes naturally producing itaconic acid as one of the top 12 value-added platform chemicals derived from biomass with multiple application areas (Geiser et al., 2014; Olicón-Hernández et al., 2019; Werpy and Petersen, 2004; Wierckx et al., 2020). Besides being used for paper, paint and fiber production, itaconic acid is also of increasing interest in the medical and pharmaceutical sectors due to its anti-inflammatory properties (Michelucci et al., 2013; Mills et al., 2018; Olagnier et al., 2020). It has been discovered that human macrophages produce itaconic acid to inhibit pathogenic bacteria, however, some bacteria have evolved resistance by itaconate (ITA) degradation (Martin et al., 1961; Sasikaran et al., 2014). A similar arms race may exist between ITA-producing fungi and their natural competitors, which might serve as a simplified model for investigating these interactions. Interestingly, Guevarra and Tabuchi (1990a) identified 2-hydroxyparaconate (2-HP) and itatartarate (ITT) as two itaconate downstream products in *Ustilago* species. At that time, the application range of 2-HP and ITT was very limited (Mori and Fukamatsu, 1992). Since then the pharmaceutical relevance of ITA has grown tremendously, which has put the derivatives in a new light as potential ITA analogs and inhibitors of the ITA degradation pathway, as explored by de Witt et al. (2023). Hypothetically, fungi evolved these compounds to counteract ITA resistance, putting them one step ahead of the human immune system. Accordingly, pharmaceutical use of these compounds may support the human immune system. To confirm this hypothesis and explore potential further applications of 2-HP and ITT, high-purity substances are obligatory. In addition, the *Ustilago* platform has undergone significant development in recent years, making a drop-in production of 2-HP/ITT in an ITA production facility more feasible, provided that a strain can be obtained with sufficient production specificity.

As shown in the model host *Ustilago maydis*, 2-HP is directly synthesized from ITA via the itaconate P450 monooxygenase Cyp3 (Geiser et al., 2016a), which can be further converted to ITT, hypothetically by abiotic lactone hydrolysis. Both acids are suggested to be secreted into the medium by the major facilitator Itp1, which is also responsible for ITA export (Geiser et al., 2018; Hosseinpour Tehrani et al., 2019a). Since low pH values (pK_a_ > pH) allow the re-uptake of ITA facilitating its conversion to 2-HP (Geiser et al., 2016a; Guevarra and Tabuchi, 1990b), highly acid-tolerant *Ustilago cynodontis* is considered as the favorable production host for these downstream compounds. Production of ITA in *U. cynodontis* has already been optimized by morphological and metabolic engineering including the deletion of *fuz7* allowing yeast-like growth and *cyp3* encoding the itaconate P450 monooxygenase preventing further conversion of ITA, the heterologous overexpression of the mitochondrial tricarboxylate transporter gene *mttA* from *Aspergillus terreus* as well as the overexpression of the itaconate cluster regulator encoding gene *ria1* (Hosseinpour Tehrani et al., 2019b, 2019d). Using this engineered itaconate-overproducing strain as a basis for further metabolic engineering, we optimized 2-HP and ITT production and purified both acids in high purity to allow comprehensive characterizations. Conversion of ITA to 2-HP was restored via overproduction of Cyp3 and exchange of the native itaconate transporter Itp1 with the *A. terreus* itaconate transporter MfsA leading to an accumulation of 2-HP and ITT upon batch fermentations on glucose and glycerol. Highest 2-HP and ITT product yields were achieved in a low cell-density fed-batch fermentation using glucose as a carbon source and acidic conditions during production resulting in 13.5 ± 0.4 g L^−1^ 2-HP and 30.5 ± 0.2 g L^−1^ ITT. Both products were recovered from the fermentation supernatants according to a protocol adapted from Guevarra and Tabuchi (1990b). Quantitative nuclear magnetic resonance spectroscopy (qNMR) measured a product purity of 85.3 ± 3.7% providing the basis for exploring their pharmaceutical relevance in follow-up studies.

## 2 Results and discussion

### 2.1 Overexpression of the itaconate-oxidizing P450 monooxygenase Cyp3

**Fig. 1:**
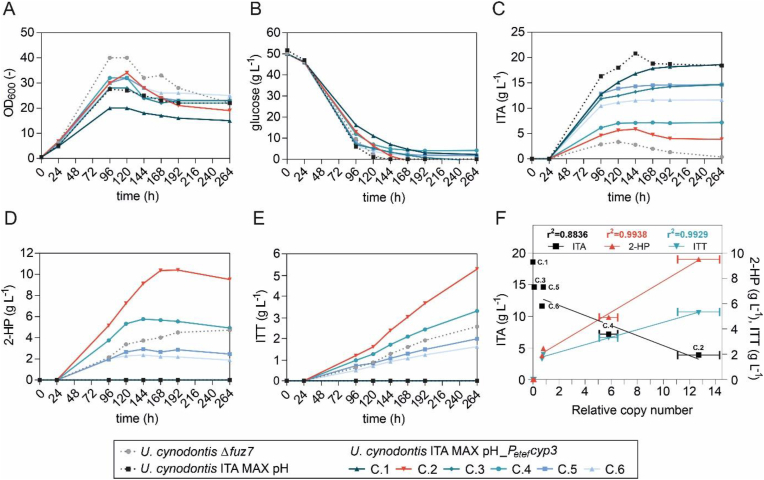
**Screening of different *U. cynodontis* ITA MAX pH_*P***_***etef***_***cyp3* clones regarding production of 2-HP and ITT as the two downstream products of ITA.** (A) Growth, (B) glucose consumption, (C) ITA production, (D) 2-HP production and (E) ITT production of the different clones and the reference strains during cultivation in shake flasks in MTM medium with 15 mM NH_4_Cl, 30 mM MES pH 6.5, and 50 g L^−1^ glucose at 30 °C and 200 rpm (n = 1 biological replicate). (F) Correlation between relative *cyp3* copy numbers and product titers of ITA, 2-HP and ITT. Copy numbers of *cyp3* were calculated via qPCR in relation to *tad1* and *rdo1* genes (both single copy) from biological triplicates measured as technical duplicates. The linear fit is shown for the *cyp3*-bearing clones.

Since 2-HP and ITT are metabolically linked to ITA, the previously engineered itaconate-overproducing *U. cynodontis* NBRC9727 Δ*fuz7* Δ*cyp3 P*_*etef*_*mttA P*_*ria1*_*ria1* (referred to as *U. cynodontis* ITA MAX pH) served as the basis for strain development. Alternatively, both modifications *P*_*etef*_*mttA* and *P*_*ria1*_*ria1* could have been re-introduced in the Δ*fuz7* strain. However, due to the random integration of the constructs in the currently best-performing ITA producer, this would not result in the same strain background. Production of the derivatives by *U. cynodontis* ITA MAX pH was restored through the *P*_*etef*_-driven overexpression of the itaconate P450 monooxygenase Cyp3, aiming at the conversion of ITA to 2-HP. To this end, the plasmid designated as *P*_*etef*_*cyp3* was randomly inserted into the genome of *U. cynodontis* ITA MAX pH. The corresponding plasmid map is shown in Fig. S1. Given that random integration of constructs into the genome often results in multi-copy and/or ectopic insertions, several clones of *U. cynodontis* ITA MAX pH_*P*_*etef*_*cyp3* were characterized to identify the best 2-HP/ITT producer. From this initial screening (Fig. S2), six clones were subjected to further characterization regarding 2-HP and ITT production in comparison to the progenitor strain as negative control and *U. cynodontis* Δ*fuz7* as positive control (Fig. 1).

Engineered strains exhibited differences in growth in comparison to the Δ*fuz7* control (Fig. 1A). This can be traced back to the overexpression of *P*_*etef*_*mttA* as previously shown by Hosseinpou r Tehrani et al. (2019b). The constitutive promotor *P*_*etef*_ leads to high expression rates already during the growth phase (Zambanini et al., 2017), likely causing MttA to export *cis*-aconitate from the mitochondria before the onset of production of ITA and its derivatives. The *P*_*etef*_*cyp3* overexpression does not seem to have an additional negative impact on growth, with all strains except clone 1 showing similar OD_600_ values and glucose consumption rates as the progenitor strain *U. cynodontis* ITA MAX pH (Fig. 1A and B). In contrast, the strains significantly differed from each other regarding ITA, 2-HP and ITT production (Fig. 1C–E). Clone 2 achieved the highest 2-HP and ITT product titers, which were approximately two-fold higher than the wildtype reference *U. cynodontis* Δ*fuz7* (Fig. 1D and E) (Hosseinpour Tehrani et al., 2019b). The combined final titers of ITA, 2-HP and ITT of clone 2 add up to 18.7 g L^−1^, an amount that corresponds well with the production of 18.4 g L^−1^ ITA by the reference itaconate-producing strain. The second-best performance regarding 2-HP and ITT production was detected for clone 4 with product titers similar to those of the Δ*fuz7* control, whereby the two clones C1 and C3 showed no conversion of ITA to 2-HP and ITT at all. To investigate whether the variation in product titers can be attributed to differences in the copy number of *P*_*etef*_*cyp3*, a qPCR analysis was performed. The gene copy number of *cyp3* relative to two reference genes *tad1* and *rdo1* (both single copy) strongly correlated with the level of conversion of ITA to 2-HP and ITT for all tested *cyp3*-bearing clones resulting in decreasing ITA (r^2^ = 0.8836) and increasing 2-HP (r^2^ = 0.9938) and ITT (r^2^ = 0.9929) titers with increasing *cyp3* copies (Fig. 1F).

Based on these results, clone 2 harboring approximately 13 *cyp3* copies was selected as the best 2-HP/ITT producer for the following experiments, henceforth named *U. cynodontis* 2-HP. However, undesired itaconate accumulation reaching a maximum concentration of 5.9 g L^−1^ at 144 h and a final concentration of 3.9 g L^−1^ was observed. Apparently, Cyp3 activity poses a bottleneck in the conversion of ITA to its derivatives, even for this best-performing strain. Consequently, we introduced the derivatives specificity as a further KPI in addition to yield, titer, and rate, which is defined as the percentage of the combined concentrations of 2-HP and ITT over the total acid production. This derivatives specificity is a key factor for later downstream processing, which was a target for optimization in this study. 2-HP and ITT can be converted into each other by lactonization or saponification (Guevarra and Tabuchi, 1990b), but ITA will remain as undesired contaminant upon purification.

### 2.2 Glycerol as an alternative carbon source for 2-HP and ITT production

**Fig. 2:**
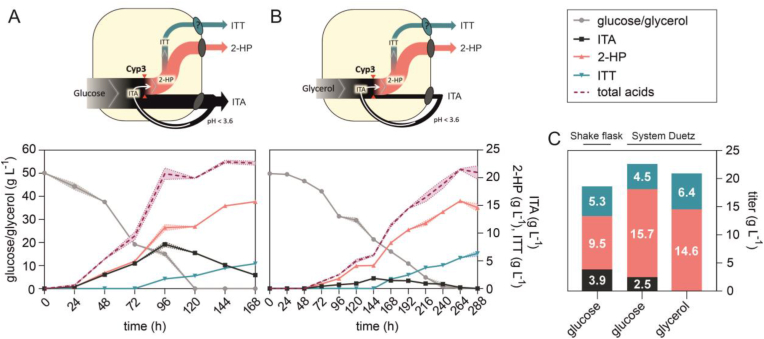
**System Duetz microcultivations of *U. cynodontis* 2-HP on two different carbon sources**. Cultivations were performed in System Duetz plates in MTM medium with 15 mM NH_4_Cl, 30 mM MES pH 6.5, and (A) 50 g L^−1^ glucose or (B) 50 g L^−1^ glycerol at 30 °C and 200 rpm (n = 2 biological replicates). For (A) and (B) glucose/glycerol consumption was plotted on the left y-axis, while ITA, 2-HP and ITT titers were plotted on the right y-axis. (C) Mean values of final product titers for direct comparison of different cultures. Product titers obtained on glucose in shake flask cultivations from Fig. 1 are shown as well for comparison. Typically, a higher OTR is achieved in System Duetz microcultivations compared to shake flask cultivations with a 10% filling volume.

*Ustilaginaceae* have a slower metabolism on glycerol than on glucose, potentially preventing an ITA overflow caused by the rate-limiting Cyp3-catalyzed conversion of ITA to 2-HP and ITT as illustrated in Fig. 2A and B. With the aim of minimizing extracellular ITA accumulation, cultivations on glycerol as an alternative carbon source were directly compared to glucose-based cultivations (Fig. 2A–C).

Indeed, accumulation of ITA was strongly reduced on glycerol, with its concentration remaining below 1.9 ± 0.1 g L^−1^ throughout the entire cultivation and a complete conversion to 2-HP and ITT after 288 h resulting in a derivatives specificity of 100 ± 0.0% (Fig. 2B). Although glucose yielded slightly more total acid production, the specificity of derivatives production was only 89 ± 0.2%. Final cumulative product titers of 2-HP and ITT were even slightly higher on glycerol than on glucose (Fig. 2C), but the high derivatives specificity did come at the expense of a lower productivity, which was reduced from 0.12 ± 0.00 g L^−1^ h^−1^ on glucose to 0.07 ± 0.00 g L^−1^ h^−1^ on glycerol. The substrate-to-product yield was comparable on both carbon sources, amounting to 0.40 ± 0.01 and 0.42 ± 0.02 g_2-HP + ITT_ g_substrate_^−1^ on glucose and glycerol, respectively.

### 2.3 Lab-scale fermentation for production of 2-HP and ITT from glycerol

**Fig. 3:**
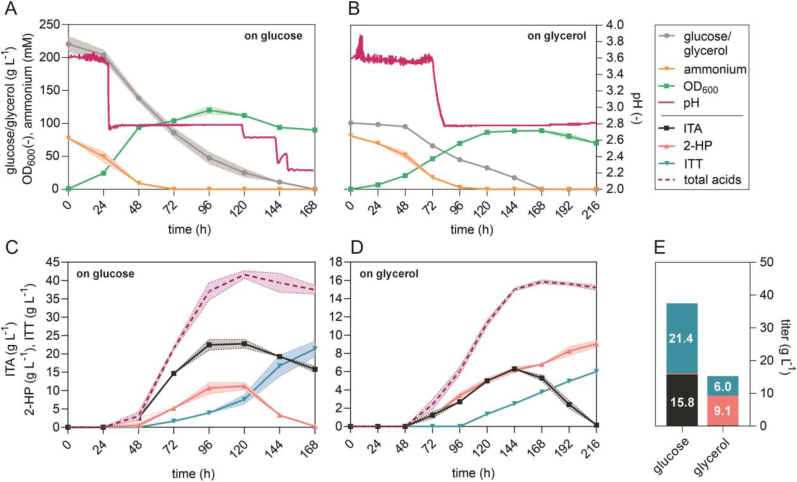
**High cell-density batch fermentations of *U. cynodontis* 2-HP on two different carbon sources.** Time course of carbon source consumption, ammonium concentrations, growth and pH during fermentation in a bioreactor containing 1 L batch medium with 75 mM NH_4_Cl and (A) 200 g L^−1^ glucose or (B) 100 g L^−1^ glycerol as carbon source (n = 2 biological replicates). Concentrations for glucose, glycerol and ammonium as well as growth as OD_600_ were plotted on the left y-axis, while pH values were plotted on the right y-axis. Corresponding product titers of ITA, 2-HP and ITT are shown in (C) for glucose-based cultivation and in (D) for glycerol-based cultivation. (E) Mean values of final product titers for direct comparison of different carbon sources. The pH was controlled by automatic titration with 5 M NaOH. After approximately 24 h (A) and 72 h (B), the pH was allowed to drop from pH 3.6 to pH 2.8 through the production of ITA and its two derivatives. Further pH reductions during the fermentation on glucose were performed by the manual addition of 1 M HCl.

High cell-density batch fermentations with a working volume of 1 L were performed on glycerol (Fig. 3B and D) and on glucose as a control (Fig. 3A and C) in order to evaluate 2-HP and ITT production at a larger scale, and to obtain larger volumes for initial downstream process development. In all fermentations, the pH was controlled at 3.6 during the initial biomass production phase, and then allowed to drop to 2.8 through the production of the organic acids (Fig. 3A and B). In the glucose fermentation, the pH was further lowered to 2.2 after 120 h in an attempt to further stimulate ITA re-uptake and conversion.

The batch fermentation on glucose resulted in the production of a mixture of all three metabolites ITA, 2-HP and ITT (Fig. 3C). The highest 2-HP concentration of 11.2 ± 1.0 g L^−1^ was reached after 120 h, but it was subsequently converted almost completely to ITT. The final ITT concentration was about 21.4 ± 1.5 g L^−1^. A total of 15.8 ± 0.9 g L^−1^ of ITA was not converted into the other products, resulting in a derivatives specificity of only 58 ± 2.6% (Fig. 3E). Even further decreased pH values, which should facilitate ITA re-uptake, did not result in a complete conversion of ITA to its two downstream products. It is important to note that the equilibrium constant between 2-HP and ITT is theoretically influenced by pH with lower pH values shifting the equilibrium towards 2-HP (Fig. S3 & Guevarra and Tabuchi (1990a)). Since the reverse reaction occurred here, we assume biotic effects to be predominant.

Under this high cell-density condition with 200 g L^−1^ glucose, the ITA production rate is apparently too high resulting in an overflow caused by the rate-limiting conversion of ITA to 2-HP and ITT (cf. Fig. 2). However, on glycerol, ITA accumulated to a much lesser extent and reached its maximum of only 6.3 ± 0.1 g L^−1^ after 144 h (Fig. 3D). In contrast to the batch fermentation with glucose, the produced ITA was completely converted to 2-HP and further to ITT yielding 99 ± 0.3% derivatives specificity. A similar production pattern was previously observed in System Duetz cultivation (Fig. 2). The glycerol fermentation resulted in an ITA-free supernatant with 9.1 ± 0.4 g L^−1^ 2-HP and 6.0 ± 0.0 g L^−1^ ITT (Fig. 3E), making the subsequent purification steps much easier.

Noteworthy, the final cumulative product titers (21.7 ± 1.5 g L^−1^ on glucose and 15.1 ± 0.3 g L^−1^ on glycerol), yields (0.10 ± 0.00 g_2-HP + ITT_ g_glucose_^−1^ and 0.15 ± 0.00 g_2-HP + ITT_ g_glycerol_^−1^) as well as the volumetric productivity (0.13 ± 0.01 g L^−1^ h^−1^ on glucose 0.07 ± 0.00 g L^−1^ h^−1^ on glycerol) were reduced during the batch fermentations in comparison to System Duetz microcultivations. This is likely caused by differences in the pH of the cultures, which was controlled in the bioreactors and not in the shaken cultures. Differences in the initial NH_4_Cl concentrations in System Duetz cultivations (15 mM) and batch fermentations (75 mM) as well as in the dissolved oxygen might also contribute to the different product KPIs.

Notwithstanding these differences, the production of larger volumes of glycerol batch fermentations enabled the purification 2-HP and ITT. Of note, these compounds are not commercially available, and only very small amounts of chemically synthesized 2-HP of unknown purity were thus far available as analytical standard (Geiser et al., 2016a). Both 2-HP and ITT were successfully obtained from the glycerol fermentation supernatant following a protocol adapted from Guevarra and Tabuchi (1990b) (Fig. S4). The obtained products were subsequently characterized by NMR analysis (^1^H, ^13^C, and quantitative) (Fig. S5), elemental analysis (Table S1), gas chromatography coupled to time-of-flight mass spectrometry (GC-ToF-MS) and Dilute-and-Shoot Flow-Injection-Analysis Tandem Mass Spectrometry (DS-FIA-MS/MS) (Reiter et al., 2022).

### 2.4 Strain engineering to enhance derivatives specificity

**Fig. 4:**
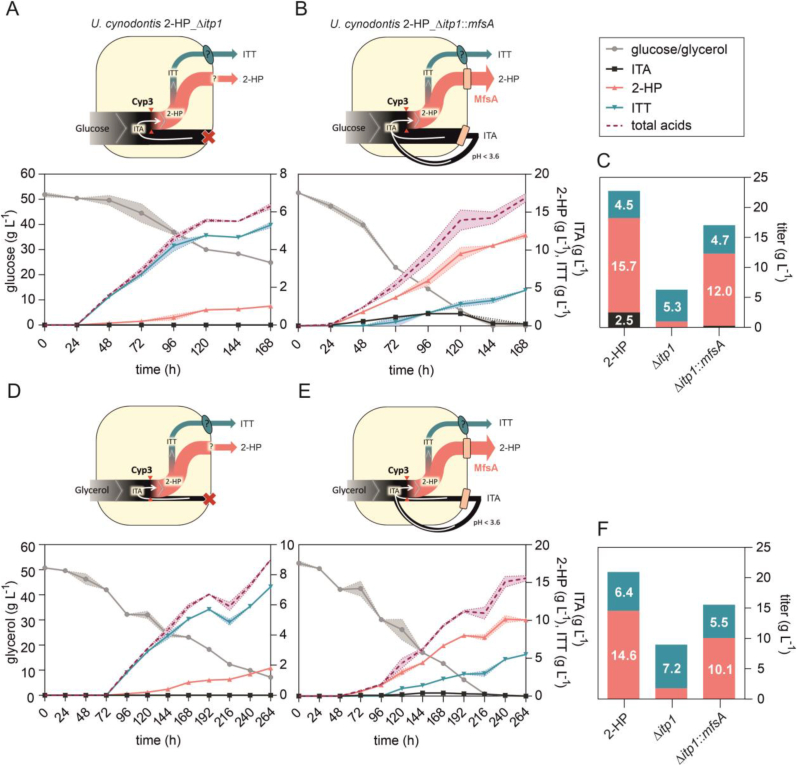
**System Duetz microcultivations of *U. cynodontis* 2-HP_Δ*itp1* and *U. cynodontis* 2-HP_Δ*itp1*::*mfsA* (henceforth named *U. cynodontis* 2-HP MfsA) on two different carbon sources for analyzing ITA, 2-HP and ITT production**. Cultivations were performed in System Duetz plates in MTM medium with 15 mM NH_4_Cl, 30 mM MES pH 6.5, and (A and B) 50 g L^−1^ glucose or (D and E) 50 g L^−1^ glycerol at 30 °C and 200 rpm (n = 2 biological replicates). Cultivations of *U. cynodontis* 2-HP_Δ*itp1* (A and D) and *U. cynodontis* 2-HP MfsA (B and E). (C and F) Mean values of final product titers for direct comparison of the different strains. Product titers obtained for *U. cynodontis* 2-HP shown in Fig. 2C are again displayed for comparison. For all time courses, carbon source consumption was plotted on the left y-axis, while ITA, 2-HP and ITT titers were plotted on the right y-axis.

The reduced metabolism on glycerol was found to significantly increase the specificity of 2-HP and ITT over ITA, thus enabling an efficient downstream processing. However, the slow glycerol metabolism came at the expense of a lower productivity compared to that achieved with glucose. To increase product specificity on glucose and thus achieve a balance between high productivity and specificity, we investigated the effects of additional metabolic engineering approaches (Fig. 4). The first approach was the deletion of the *itp1* itaconate transporter gene. At neutral intracellular pH, itaconate mainly exists in its double-deprotonated form, which is not able to pass the membrane by passive diffusion. Deletion of this transporter should therefore increase the intracellular concentration of itaconate and thus its conversion to the products of interest. Itp1 was also exchanged with the heterologous itaconate transporter MfsA from *A. terreus*, which was described to have a higher affinity for 2-HP export than Itp1 (Hosseinpour Tehrani et al., 2019a). The corresponding plasmid map is shown in Fig. S6.

The Δ*itp1* strain was not able to secrete ITA anymore throughout the entire cultivation, and the 2-HP concentration was also significantly reduced (Fig. 4A). This confirmed its role as ITA and 2-HP exporter as described for *U. maydis*, although the Δ*itp1* mutant of this strain produced 0.7 ± 0.1 g L^−1^ ITA (Hosseinpour Tehrani et al., 2019b). This mutant even showed an increased 2-HP titer in ITA feeding experiments, which points towards an additional 2-HP transport mechanism in *U. maydis* under certain conditions, which is apparently absent in *U. cynodontis*. However, in that previous manuscript ITT could not be quantified due to a lack of a suitable analytical technique and standard. With the produced Na-ITT standard, this was now addressed. Interestingly, the measured ITT concentration of 5.3 ± 0.1 g L^−1^ was higher than the one obtained for the control with intact *itp1* gene (Fig. 4C), which strongly suggests the existence of a different transport system for ITT. However, overall derivatives titers were greatly reduced and the knockout also strongly affected substrate uptake rates, likely as a result of the stress of intracellular ITA and 2-HP accumulation. This was ameliorated by the complementation with *mfsA.* The exchange of *itp1* by *mfsA* in the *U. cynodontis* 2-HP MfsA strain reduced the maximum ITA accumulation from 8.0 ± 0.42 g L^−1^ (Fig. 2A) to 1.6 ± 0.28 g L^−1^ (Fig. 4B). This corresponds to a reduction of approximately 80 ± 0.7% compared to the progenitor strain *U. cynodontis* 2-HP, resulting in an increased derivatives specificity of approximately 99 ± 0.3%. Analogous to the Δ*itp1* modification, a reduced transport of ITA via MfsA is assumed to result in higher intracellular titers for enhanced conversion into 2-HP and ITT, but this accumulation is less severe. Consequently, there was a greater cumulative production of the derivatives to 16.7 ± 0.3 g L^−1^ (Fig. 4C), although titers of the two downstream products were reduced compared to the progenitor with Itp1 transporter. Hence, the improved derivatives specificity came at the expense of a reduced substrate-to-product yield (0.40 ± 0.01 and 0.33 ± 0.01 g_2-HP + ITT_ g_glucose_^−1^ for *U. cynodontis* 2-HP and *U. cynodontis* 2-HP MfsA, respectively) and a reduced volumetric productivity of 0.10 ± 0.00 g L^−1^ h^−1^ (Fig. 4A). Here, one can assume that elevated intracellular ITA concentrations may favor its degradation limiting further conversion. Although an ITA degradation pathway has not been described for *Ustilago*, decreasing ITA concentrations have been observed in *U. maydis* during prolonged cultivations (Geiser et al., 2016a; Hosseinpour Tehrani et al., 2019c).Likely, the degradation occurs through a similar pathway as described for *A. terreus* (Chen et al., 2016) where ITA is CoA-activated, converted to citramalyl-CoA, and then cleaved into acetyl-CoA and pyruvate. Early reports speculated on citramalyl-CoA as an intermediate of the 2-HP biosynthetic pathway (Guevarra and Tabuchi, 1990a), but this has since been disproven (Geiser et al., 2016a) and hence 2-HP synthesis and ITA degradation occurs via two separate pathways. Alternatively, it might also be possible that the higher intracellular ITA concentration reduces production efficiencies. The latter seems likely, given that the Δ*itp1* strain has a greatly reduced glucose uptake rate.

Additionally, System Duetz microcultivations of these strains were also performed on glycerol, showing analogous behavior compared to glucose (Fig. 4D and E). Deletion of *itp1* caused a strong reduction in ITA and 2-HP accumulation in comparison to the progenitor strain *U. cynodontis* 2-HP (Fig. 4F), while ITT levels were increased. Related to glucose as carbon source, performance of *U. cynodontis* 2-HP MfsA on glycerol was slightly worse in terms of the achieved yield (0.30 ± 0.01 g_2-HP + ITT_ g_glucose_^−1^) and the calculated productivity (0.06 ± 0.00 g L^−1^ h^−1^), favoring cultivation of this improved strain on glucose for specific 2-HP and ITT production in up-scaled fermentations.

**Fig. 5:**
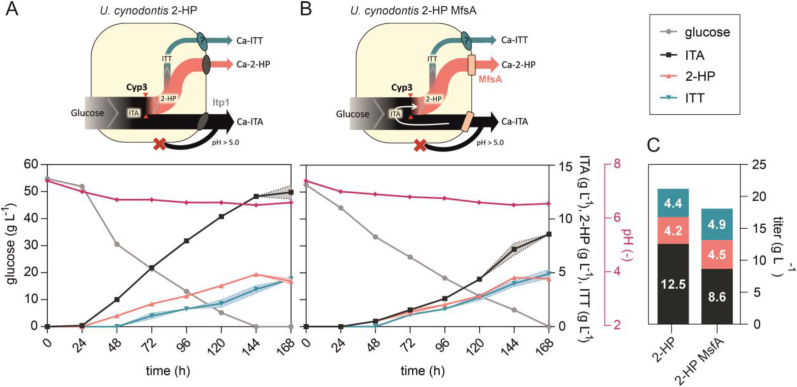
**System Duetz microcultivations of *U. cynodontis* 2-HP and *U. cynodontis* 2-HP MfsA on glucose using CaCO**_**3**_**as buffer**. (A and B) Cultivations were performed in System Duetz plates in MTM medium with 15 mM NH_4_Cl, 33 g L^−1^ CaCO_3_ pH 7.4, and 50 g L^−1^ glucose at 30 °C and 200 rpm (n = 2 biological duplicates). (C) Mean values of final product titers for direct comparison of the different strains.

Besides a reduction of ITA export, the re-uptake of ITA is also a possible factor that can contribute to 2-HP and ITT production (Geiser et al., 2016a), especially in later production stages when ITA production slows down. To further characterize this effect, additional System Duetz microcultivations were performed with CaCO_3_ as a buffer (Fig. 5). This should prevent ITA re-uptake by avoiding the formation of fully protonated ITA (Hosseinpour Tehrani et al., 2019c; Zambanini et al., 2016b), which may consequently allow to more precisely conclude on initial metabolic fluxes. In addition, calcium carbonate should reduce product inhibition and weak acid stress and thus may allow to reach higher 2-HP and ITT titers.

Cultivation of *U. cynodontis* 2-HP showed similar cumulative end titers of ITA, 2-HP and ITT when using MES (Fig. 2C) or CaCO_3_ (Fig. 5C) as buffer component. For CaCO_3_, all product titers increased continuously throughout the cultivation, except for a slight decrease in 2-HP at the end of the cultivation. Such product formation was not observed during cultivation with MES buffer due to the re-uptake and further conversion of ITA facilitated by the acidic pH values, as shown in Fig. S7. The conversion of ITA to its downstream products was markedly reduced in the presence of CaCO_3_, which prevents acidification of the media (Fig. 5A and B). This demonstrates a major contribution of the re-uptake of protonated ITA to 2-HP and ITT production (Fig. 2A and C and Fig. 5A and C). Compared with the progenitor strain *U. cynodontis* 2-HP pH, cultivation of *U. cynodontis* 2-HP MfsA on glucose with CaCO_3_ buffer showed reduced absolute and relative accumulation of ITA, while more 2-HP and ITT was secreted. This supports the hypothesis that MfsA has a higher affinity for 2-HP and lower affinity for ITA (Hosseinpour Tehrani et al., 2019a) and it further suggests that it also facilitates 10.13039/100004373ITT transportation (Fig. 4C and 5B and C). The fact that these differences are observed with the CaCO_3_ buffer proves that the increased specificity of derivatives production is caused by MfsA and not by altered ITA re-uptake dynamics. However, ITA is reduced but not entirely abolished, and therefore a combination of specificity-driving strategies is needed to achieve full conversion.

### 2.5 Bioreactor cultivations on glucose

The exchange of *itp1* with *mfsA* resulted in a substantially increased derivatives specificity, which was further enhanced in cultures with a low pH facilitating ITA re-uptake (Fig. 4). We therefore sought to leverage these strategies to maintain the high productivity on glucose compared to glycerol while still achieving full conversion in a high cell-density batch fermentation.

**Fig. 6:**
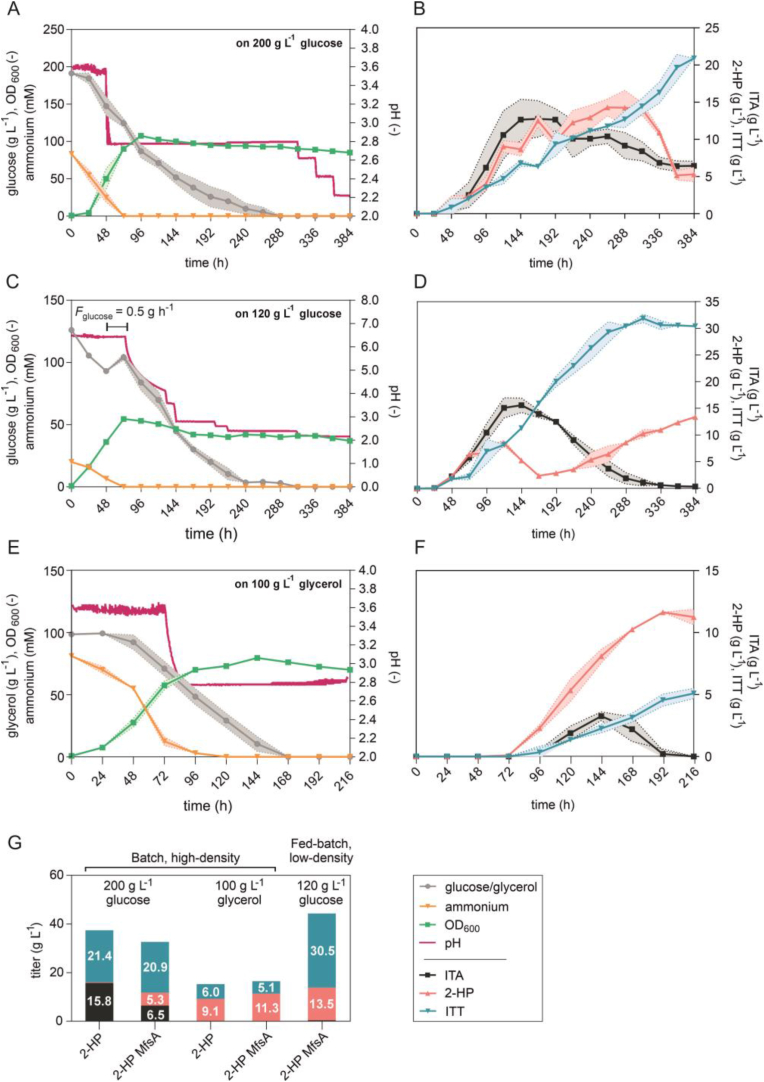
**Fermentation approaches of *U. cynodontis* 2-HP MfsA to optimize 2-HP and ITT production.** (A) High cell-density batch fermentation with 75 mM NH_4_Cl and 200 g L^−1^ glucose with corresponding product titers of ITA, 2-HP and ITT shown in (B). (C) Low cell-density fed-batch fermentation with 15 mM NH_4_Cl and 120 g L^−1^ glucose including an additional 12 g glucose fed with corresponding product titers of ITA, 2-HP and ITT shown in (D). (E) High cell-density batch fermentation with 75 mM NH_4_Cl 100 g L^−1^ glycerol with corresponding product titers of ITA, 2-HP and ITT shown in (F). In case of (A, C and E), carbon source consumption, ammonium concentration and growth were plotted on the left y-axis, while pH values were plotted on the right y-axis. The pH was controlled by automatic titration with 5 M NaOH. After approximately 48 h (A) and 72 h (C and E), the pH was allowed to drop from pH 3.6 to pH 2.8 (A and E) and from 6.5 to 3.6 (C) through the production of ITA and its two derivatives. Further pH reductions during the fermentations on glucose were performed by the manual addition of 1 M HCl. (F) Mean values of final product titers for all performed lab-scale fermentations of strain *U. cynodontis* 2-HP and *U. cynodontis* 2-HP MfsA (n = 2 biological replicates).

Previous high cell-density batch fermentation of *U. cynodontis* 2-HP resulted in the production of a mixture of all three metabolites (Fig. 3). Analogous batch fermentation of the newly generated *U. cynodontis* 2-HP MfsA harboring the Δ*itp1*:*mfsA* modification showed a similar production pattern, but with a 69 ± 0.5% overall reduction in extracellular ITA accumulation due to the transporter exchange. However, the ITA is not fully converted even after prolonged incubation and a further decrease of pH (Fig. 3C and 6A, B and G). Of note, once the pH value was adjusted to 2.6, a strong decrease in the 2-HP concentration was observed. This decrease most likely resulted from the re-uptake of 2-HP at this pH value, as it is around the pK_a_ value of 2-HP (2.78) (Guevarra and Tabuchi, 1990a). While *U. cynodontis* 2-HP metabolized all glucose within 168 h, *U. cynodontis* 2-HP MfsA required an additional 120 h for the complete conversion. The strain also showed a much longer lag phase, indicating that the transporter exchange caused significant stress to the production host especially in this high glucose concentration. However, the final cumulative product titers of 2-HP and ITT were higher with 26.2 ± 0.8 g L^−1^ compared to *U. cynodontis* 2-HP, resulting in an increased derivatives yield (Table 1).

**Table 1:** **KPIs of the different fermentation approaches, calculated for the endpoint titers**.

Strain	Fermentation	Product	Titer (g L^−1^)	Yield (g_product_ g_substrate_^−1^)	Productivity (g L^−1^ h^−1^)	Derivatives specificity (%)
2-HP	200 g L^−1^ glucose	2-HP + ITT	21.7 ± 1.5	0.10 ± 0.00	0.13 ± 0.01	58 ± 2.6%
		2-HP	0.3 ± 0.1	0.00 ± 0.00	0.00 ± 0.00	
		ITT	21.4 ± 1.5	0.10 ± 0.00	0.13 ± 0.01	
	100 g L^−1^ glycerol	2-HP + ITT	15.1 ± 0.3	0.15 ± 0.00	0.07 ± 0.00	99 ± 0.3%
		2-HP	9.1 ± 0.3	0.10 ± 0.00	0.04 ± 0.00	
		ITT	6.0 ± 0.0	0.06 ± 0.00	0.03 ± 0.00	
2-HP MfsA	200 g L^−1^ glucose	2-HP + ITT	26.2 ± 0.8	0.14 ± 0.00	0.07 ± 0.00	80 ± 0.6%
		2-HP	5.3 ± 0.7	0.03 ± 0.00	0.01 ± 0.00	
		ITT	20.9 ± 0.1	0.11 ± 0.00	0.05 ± 0.00	
	120 g L^−1^ glucose	2-HP + ITT	44.0 ± 0.2	0.32 ± 0.01	0.11 ± 0.00	99 ± 0.2%
		2-HP	13.5 ± 0.4	0.10 ± 0.00	0.04 ± 0.00	
		ITT	30.5 ± 0.2	0.22 ± 0.01	0.08 ± 0.00	
	100 g L^−1^ glycerol	2-HP + ITT	16.4 ± 0.2	0.17 ± 0.00	0.08 ± 0.00	100 ± 0.0%
		2-HP	11.3 ± 0.5	0.11 ± 0.01	0.05 ± 0.00	
		ITT	5.1 ± 0.3	0.05 ± 0.00	0.02 ± 0.00	

Since the high cell-density batch fermentations on glucose did not result in the production of pure 2-HP and ITT, a low cell-density fed-batch fermentation with a reduced glucose starting concentration was assessed next (Fig. 6C and D). To further reduce stress in the initial biomass production phase, the starting pH value was set to 6.5 (Niehoff et al., 2023). Afterwards, the pH value was allowed to drop through the production of ITA and its two downstream products 2-HP and ITT. However, since the pH value only dropped slowly, it was manually adjusted to 3.6 after 130 h by adding HCl. ITA accumulated up to 15.7 ± 1.3 g L^−1^, comparable to the previous high cell-density batch fermentation (Fig. 3C and 6C, D and G). The high ITA accumulation may be due to the elevated pH value during the initial biomass production phase, preventing secreted ITA from re-entering the cells. Due to the already high ITA concentration, glucose was fed only for a short period of 24 h, and the pH value was further decreased to facilitate the re-uptake of ITA. ITA was continuously converted into its downstream products until it was completely depleted at the end of the fermentation accounting for a derivatives specificity for 2-HP and ITT of 99 ± 0.2%. After 288 h of fermentation, the ITT concentration remained constant, while the 2-HP concentration increased despite a pH value of 2.2, which theoretically allows 2-HP to be re-uptaken and transformed into ITT. This suggests that a threshold of 30.5 ± 0.4 g L^−1^ ITT may exists, beyond which the ITT production rate is strongly reduced, possibly due to feedback inhibition, or precipitation. The final broth contained more ITT than 2-HP with a combined product titer of 44.0 ± 0.2 g L^−1^. Guevarra and Tabuchi (1990b) reported a higher 2-HP titer of 30 g L^−1^, but a lower ITT titer of 25 g L^−1^ with the wildtype progenitor of the engineered strains described in this study, but we were unable to reproduce these results due to strong filamentous growth and lower productivities of this non-engineered strain. Perhaps this can be attributed to minor differences in cultivation conditions, such as the use of urea as alternative nitrogen source. In previous studies with *U. cynodontis* Δ*fuz7* (Hosseinpour Tehrani et al. (2019d) and *U. maydis* MB215 (Geiser et al., 2016a), 2-HP production was accompanied by accumulation of high concentrations of ITA, while ITT was typically not analyzed. This highlights the requirement for strain and process optimization to obtain a high 2-HP and ITT derivatives specificity as successfully done in this work. Additionally, for all previously published data potential inaccuracies deriving from standards with unknown purity should be taken into account, further complicating direct comparisons.

To evaluate the derivatives specificity of 2-HP and ITT over ITA across the cultivation time on glycerol as a carbon source, an additional fermentation was performed with the newly engineered *U. cynodontis* 2-HP MfsA (Fig. 6E, F, and G). Accumulation of ITA during fermentation was substantially lower than on glucose reaching a maximum value of 3.3 ± 0.4 g L^−1^, but final cumulative product titers of 2-HP and ITT were markedly lower during cultivation of *U. cynodontis* 2-HP MfsA on glycerol as well. When compared to the progenitor strain's performance on glycerol, the maximum ITA accumulation was reduced from 6.3 ± 0.14 g L^−1^ (Fig. 3D) to 3.25 ± 0.35 g L^−1^ (Fig. 6F). This corresponds to a reduction of approximately 48 ± 0.5%. Moreover, whereas product titers of 2-HP and ITT were almost comparable for *U. cynodontis* 2-HP pH and *U. cynodontis* 2-HP MfsA on glycerol, the latter exhibited a faster conversion of ITA to 2-HP and ITT, achieving complete conversion in just 192 h, whereas the progenitor strain required 216 h (Fig. 3, Fig. 6F). The growth of both strains was similar, with ammonium depletion occurring after 120 h and glycerol depletion after 168 h in both fermentations (Fig. 3, Fig. 6). This is in contrast to the glucose cultures, where the transporter exchange caused decreased growth and production rates. Likely, the overall reduced metabolic fluxes on glycerol led to a better balancing of substrate uptake, ITA production, and product secretion rates, preventing excessive intracellular ITA accumulation while still improving 2-HP and ITT specificity.

## 3 Conclusion

This study explores different strategies to increase derivatives specificity for the production of 2-HP and ITT over ITA, including process parameters such as substrate and pH, as well as strain engineering focused on Cyp3 expression and product export. This multifactorial approach highlights the challenges and solutions related to the specific production of a certain compound in a pathway with multiple derivatives, as is often the case with secondary metabolites and organic acids (Konishi and Makino, 2018; Yang et al., 2020). A high copy number of *cyp3* was essential for efficient 2-HP production, but still posed a bottleneck even in the best-performing strain. The linear correlation between copy number of the *P*_*etef*_*cyp3* cassette and production suggests that Cyp3 activity may still be boosted by further overexpression.

Knockout of the exporter gene *itp1* abolished ITA production, but with a drastic reduction in overall production. Exchange of the Itp1 transporter for MfsA from *A. terreus* increased specificity with a much smaller effect on production efficiency, but overall, strain engineering alone could not increase derivatives specificity to 100% without significantly reducing yield, titer, and rate. Specificity could be driven to 100 ± 0.0% by a combination of the transporter exchange, low pH to facilitate ITA re-uptake, and use of glycerol to reduce ITA production rate. However, the best yield, titer, and rate for 2-HP and ITT were achieved when performing a low cell-density fed-batch fermentation on glucose combining high product titers (44.0 ± 0.2 g L^−1^, high derivatives specificity (99 ± 0.2%), the highest yield of 0.32 ± 0.01 g_2-HP + ITT_ g_glucose_^−1^ and one of the highest volumetric productivities (0.11 ± 0.00 g L^−1^ h^−1^) (Table 1). Nevertheless, significant amounts of ITA still accumulated during this fermentation, which may be addressed in the future by further strain engineering to optimize Cyp3 expression and activity as a main focus.

In summary, 2-HP and ITT of relatively high purity were produced, building upon the pioneering work of Guevarra and Tabuchi (1990b). This now enables the study of these ITA derivatives for potential pharmaceutical applications, in light of recent discoveries on the antibacterial relevance of their ITA precursor (de Witt et al., 2023; Li et al., 2023; O'Neill and Artyomov, 2019; Peace and O’Neill, 2022).

## 4 Materials and methods

### 4.1 Chemicals and strains

All chemicals used in this study were obtained from Sigma-Aldrich (St. Louis, USA), Thermo Fisher Scientific (Waltham, USA), or VWR (Radnor, USA) and were of analytical grade. All strains used in this work are listed in Table 2.

**Table 2:** *U. cynodontis* strains used in this study.

Strain designation	Resistance	Reference
*U. cynodontis* NBRC9727 Δ*fuz7* (162)	–	Hosseinpour Tehrani et al. (2019b)
*U. cynodontis* NBRC9727 Δ*fuz7* Δ*cyp3 P*_*etef*_*mttA P*_*ria1*_*ria1* (223)	Hyg^R^, Cbx^R^	Hosseinpour Tehrani et al. (2019b)
*U. cynodontis* NBRC9727 Δ*fuz7* Δ*cyp3 P*_*etef*_*mttA P*_*ria1*_*ria1 P*_*etef*_*cyp3* (1375)	Hyg^R^, Cbx^R^, Nat^R^	this study
*U. cynodontis* NBRC9727 Δ*fuz7* Δ*cyp3 P*_*etef*_*mttA P*_*ria1*_*ria1 P*_*etef*_*cyp3* Δ*itp1* (2694)	Hyg^R^, Cbx^R^, Nat^R^, G418^R^	this study
*U. cynodontis* NBRC9727 Δ*fuz7* Δ*cyp3 P*_*etef*_*mttA P*_*ria1*_*ria1 P*_*etef*_*cyp3* Δ*itp1*::*mfsA* (2695)	Hyg^R^, Cbx^R^, Nat^R^, G418^R^	this study

Media and culture conditions.

*U. cynodontis* strains were grown in YEPS medium containing 10 g L^−1^ yeast extract, 10 g L^−1^ peptone, and 10 g L^−1^ sucrose. For growth and production experiments, *U. cynodontis* was cultured in 30 mM MES or 33 g L^−1^ CaCO_3_ buffered screening medium according to Geiser et al. (2014) with either 50 g L^−1^ glucose or glycerol. The medium also contained 0.8 g L^−1^ NH_4_Cl, 0.2 g L^−1^ MgSO_4_·7H_2_O, 0.01 g L^−1^ FeSO_4_·7H_2_O, 0.5 g L^−1^ KH_2_PO_4_, 1 mL L^−1^ vitamin solution, and 1 mL L^−1^ trace element solution. The vitamin solution contained (per liter) 0.05 g D-biotin, 1 g D-calcium pantothenate, 1 g nicotinic acid, 25 g myo-inositol, 1 g thiamine hydrochloride, 1 g pyridoxol hydrochloride, and 0.2 g *para*-aminobenzoic acid. The trace element solution contained (per liter) 1.5 g EDTA, 0.45 g ZnSO_4_·7H_2_O, 0.10 g MnCl_2_·4H_2_O, 0.03 g CoCl_2_·6H_2_O, 0.03 g CuSO_4_·5H_2_O, 0.04 g Na_2_MoO_4_·2H_2_O, 0.45 g CaCl_2_·2H_2_O, 0.3 g FeSO_4_·7H_2_O, 0.10 g H_3_BO_3_,and 0.01 g KI. Cultivations were conducted in System Duetz plates (24 well plates, Enzyscreen, Netherlands) with a filling volume of 1.5 mL (d = 50 mm, n = 300 rpm, T = 30 °C and Φ = 80%) (Duetz et al., 2000) or in 500 mL shaking flasks with a filling volume of 50 mL (d = 25 mm, n = 200 rpm, T = 30 °C and Φ = 80%). For growth and production experiments, main cultures were inoculated to an OD_600_ of 0.5 with overnight precultures grown in the same media. When performing System Duetz cultivations, cultures were simultaneously inoculated into multiple plates. A full plate was designated as a sacrificial sample for each time point to maintain continuous oxygenations.

Controlled fed-batch cultivations were carried out in a DASGIP® Bioblock (Eppendorf, Germany), controlled using the Eppendorf DASware® control software (Eppendorf, Germany). Vessels with a total volume of 2.3 L and a working volume of 1.0 L were used. All cultivations were performed in batch medium according to Geiser et al. (2014) as described above. The medium also contained 1 g L^−1^ yeast extract (Merck Millipore, Germany) and varying concentrations of glucose, glycerol and NH_4_Cl, as indicated. The pH was controlled by automatic addition of 5 M NaOH or 1 M HCl, and the DO was controlled at 30% by a cascade mode including agitation 800–1200 rpm (0–40% DOT controller output), air flow 1–2 vvm (40–80% DOT controller output), and oxygen 21–100% (80–100% DOT controller output). The cultivation was performed at 30 °C and the bioreactor was inoculated to a final OD_600_ of 0.75 from an overnight preculture grown in screening medium according to Geiser et al. (2014) containing 50 g L^−1^ glucose or glycerol, 0.8 g L^−1^ NH_4_Cl and 100 mM MES pH 6.5. 0.5 mL Antifoam 204 (Sigma, A6426) was added in the beginning of the cultivation and subsequently every 24 h.

### 4.2 Analytical methods

When CaCO_3_ was used as a buffer, it was dissolved in a 1:1 ratio with 4 M HCl before subsequent measurements, according to the procedure described by Zambanini et al. (2016a). Identification and quantification of products and substrates in the supernatants were performed using a High Performance Liquid Chromatography (HPLC) 1260 Infinity system (Agilent, Waldbronn, Germany) equipped with an ISERA Metab AAC column 300 × 7.8 mm column (ISERA, Germany). Separation was performed using an isocratic elution program at a flow rate of 0.6 mL min^−1^ and a temperature of 40 °C with 5 mM sulfuric acid as a solvent. Detection was performed using a diode array detector (DAD) at 210 nm and a refraction index (RI) detector. All samples were filtered with Rotilabo® syringe filters (pore size 0.22 μm) and then diluted with ddH_2_O. Analytes were identified on the basis of retention time compared to corresponding standards, and data analysis was performed using the Agilent OpenLAB Data Analysis - Build 2.200.0.528 software (Agilent, Waldbronn, Germany). Ammonium concentrations in culture samples were determined by the colorimetric method described by Willis et al. (1996). In this method, 50 μL culture supernatant was mixed with 1 mL reagent (8 g sodium salicylate, 10 g trisodium phosphate, 0.125 g sodium nitroprusside), followed by rapid addition of 250 μL hypochlorite solution. After color development (at least 15 min at RT), the absorbance was measured at 685 nm using cuvettes and a spectrophotometer. Ammonium concentrations were calculated using an ammonium standard curve. Cell densities were quantified by measuring the optical density at a wavelength of 600 nm (OD_600_) using cuvettes and a spectrophotometer. Samples were diluted appropriately with the respective medium to fall within the linear measuring range of the photometer between absolute values of 0.2 and 0.4.

### 4.3 Product purification and characterization

Following the successful production of 2-HP and ITT, the corresponding fermentation broths were centrifuged at 10,000 rpm for 30–60 min using a Beckman Avanti J-25 Centrifuge from Beckman Instruments (Fullerton, USA). The cell pellet was discarded, and the resulting supernatant was filtered (pore size 0.22 μm). The products 2-HP and ITT were recovered from the fermentation supernatant through a process adapted from Guevarra and Tabuchi (1990b). The lactonization was started with the evaporation of water from the supernatant in a rotary evaporator, resulting in a syrup. Büchi® R-210 Rotavapor® Evaporator from BÜCHI Labortechnik GmbH (Essen, Germany) was used, which was equipped with a Büchi® B-491 Heating Bath and a diaphragm vacuum pump CVC 2 from VACUUBRAND (Wertheim, Germany). The temperature of the water bath was set to 50–60 °C, and the pressure was gradually decreased to approximately 60 mbar, yielding in a concentrated syrup in about 1 h. Subsequently, the temperature of the water bath was gradually raised to 90 °C, the pressure gradually reduced to the minimum of approximately 20 mbar, and the syrup was further heated for at least 6 h to lactonize ITT to 2-HP. The resulting mass was dried with the high vacuum pump TRIVAC D 4 B from Leybold (Köln, Germany) and simultaneously heated with a heating mantle to eliminate residual water over a period of about 2–3 h. The dry mass was dissolved in the same volume of hot ethyl acetate as the initial volume of supernatant. The extraction with ethyl acetate involved continuous stirring and heating (50–60 °C) for approximately 6–7 h, utilizing a reflux condenser to prevent solvent evaporation. Depending on the experiment, 2 to 5 extraction steps were performed with fresh solvent to enhance product recovery. Insoluble impurities were removed through decantation and subsequent filtration (pore size 0.22 μm). The solvent phases from the extraction steps were combined and concentrated using the rotary evaporator. The resulting volume was aliquoted into pre-weighed Eppendorf tubes, and the remaining ethyl acetate was evaporated in the Vacufuge Concentrator Model 5301 from Eppendorf (Hamburg, Germany) to obtain dry 2-HP. The tubes were reweighed to determine the final product quantity. The sodium salt of ITT was obtained by saponifying the 2-HP recovered from the fermentation supernatant, following the procedure described by Guevarra and Tabuchi (1990b). A known mass of 2-HP was dissolved in ddH_2_O to obtain a solution of known molarity. The volume of 5 M NaOH required to neutralize the acidic solution was calculated, and twice this volume was added to the 2-HP solution. The mixture was shaken and heated (60–70 °C) for approximately 10–15 min to saponify 2-HP. Finally, the Na-ITT was dried in the Vacufuge Concentrator Model 5301 from Eppendorf (Hamburg, Germany). Several analytical methods were employed for product characterization. NMR spectroscopy analyses, including ^1^H NMR, ^13^C NMR, and qNMR, were performed with the Avance DRX600 NMR Spectrometer from Bruker Corporation (Billerica, USA). For the NMR analyses, dry 2-HP was dissolved in deuterated acetone, while dry Na-ITT was dissolved in deuterated water. For qNMR, approximately 5 mg of 2-HP and a similar mass of the standard 1,3,5-trimethoxybenzene (to obtain similar molarities) were mixed and dissolved in deuterated acetone. The elemental analysis was conducted with the vario EL cube system from Elementar (Langenselbold, Germany). Small amounts of the products (2 mg) were analyzed in both CHN and O modes. The products were also analyzed by GC-ToF-MS with a method adapted from Paczia et al. (2012). The pH of the samples was adjusted to fall within the neutral range of 6.7–7.3 using a NaOH solution and the 766 Laboratory pH Meter from Knick (Berlin, Germany). Dry products were dissolved either in ddH_2_O or in 50 mM phosphate buffer with pH 7.0. Samples with volumes of 13 μL or 130 μL and with concentrations not exceeding 5 mM were prepared, shock-frozen in liquid nitrogen and stored at −20 °C. The samples were afterwards lyophilized overnight in a Christ LT-105 freeze dryer from Martin Christ Gefriertrocknungsanlagen GmbH (Osterode am Harz, Germany). Following lyophilization, the dried samples were derivatized with 50 μL MeOX (20 mg mL^−1^ O-methylhydroxylamine in pyridine) for 90 min at 30 °C and 600 rpm in a ThermoMixer from Eppendorf (Hamburg, Germany). This was followed by an incubation with 80 μL of MSTFA (N-methyl-N-(trimethylsilyl)-trifluoracetamide) for 90 min at 40 °C and 600 rpm. The analysis was conducted using an 8890N double SSL gas chromatograph from Agilent (Santa Clara, USA) equipped with a LPAL3-S15 liquid autosampler from LECO (Mönchengladbach, Germany). The gas chromatograph was coupled to a GCxGC HRT+ 4D high-resolution time of flight mass spectrometer from LECO (Mönchengladbach, Germany). A volume of 1 μL of sample was injected into a split/splitless injector at 280 °C at varying split modes. The constant helium flow was set to 1 mL min^−1^ for the active injector and column, and to 0.5 mL min^−1^ for the passive injector. For peak identification, the Retention time Index (RTI) value, the baseline noise subtracted fragmentation pattern and the fragment elemental composition were compared to an in-house m/z database JuPoD and the commercial database NIST20 (National Institute of Standards and Technology, USA). Purified products were also subjected to analysis by DS-FIA-MS/MS (Reiter et al., 2022). The obtained 2-HP and ITT were dissolved in ddH_2_O or in 50 mM phosphate buffer with pH 7.0 prior to analysis. Accurate mass spectra were acquired using the ESI-QqToF MS TripleTOF6600 from AB Sciex (Darmstadt, Germany). An Agilent 1100 system, along with an Agilent 1260 Infinity II Multisampler from Agilent Technologies (Waldbronn, Germany), coupled to an ESI-QqQ API4000 from AB Sciex (Darmstadt, Germany) was employed for the analysis.

### 4.4 Plasmid cloning and strain engineering

Plasmids were constructed via Gibson assembly (Gibson et al., 2009) employing the NEBuilder® HiFi DNA Assembly Cloning Kit (New England Biolabs (NEB), Ipswich, MA, USA). DNA oligonucleotides were purchased from Eurofins Genomics (Ebersberg, Germany), and Q5® High-Fidelity DNA Polymerase (NEB) served as the polymerase. Details about the utilized primers and plasmids are shown in Table 3 and Table S2. Standard cloning and plasmid maintenance were carried out using competent *E. coli* DH5α or PIR2 cells according to Sambrook and Russell (2006). Plasmids were confirmed by polymerase chain reaction, restriction or sequencing. For the generation of protoplasts, transformation, and isolation of genomic DNA of *U. cynodontis* NBRC9727, protocols according to Brachmann et al. (2004) were used. For the integration of *P*_*etef*_*cyp3*, the plasmid was linearized with *Fsp*I and integrated randomly into the genome. For the deletion of *itp1*, homologous recombination with 1000 bp flanking regions including FRT-sites and a geneticin G418 resistance cassette were used. For the exchange of *itp1* with *mfsA*, homologous recombination with 1000 bp flanking regions and a geneticin G418 resistance cassette were used. Successful integration, deletion and exchange were confirmed by PCR.

**Table 3:** Plasmids used in this study.

Plasmid	Description	Reference
*P*_*etef*_05074_Cbx	Constitutive *P*_*etef*_ promotor, *cyp3* gene from *U. maydis* MB215, Cbx^R^	Geiser et al. (2016b)
pUMa3479	FRTm2-NatR-FRTm2 cassette	Dr. K. Schipper, Heinrich-Heine University Düsseldorf, Germany
pJET1.2/blunt	Ori ColE1, Amp^R^	Thermo Scientific, Germany
pUMa3414	FRTm7-NatR-FRTm7 cassette	Dr. K. Schipper, Heinrich-Heine University Düsseldorf, Germany
*P*_*etef*_*gfp*_G418	Constitutive *P*_*etef*_ promotor, *gfp* gene, G418^R^	(Przybilla, Roxense BioSC)
*P*_*etef*_*AT_mfsA*_Cbx	Constitutive *P*_*etef*_ promotor, dicodon-optimized version of *A. terreus* ATEG 09972 (mfsA), Cbx^R^	Hosseinpour Tehrani et al. (2019a)
*P*_*etef*_*cyp3*_Nat	Constitutive *P*_*etef*_ promotor, *cyp3* gene from *U. maydis* MB215, Nat^R^, FRTm2	this study
Δ*itp1*_G418	Deletion of the *itp1* gene in *U. cynodontis* 2-HP, G418^R^, FRTm7	this study
Δ*itp1*::*mfsA*_G418	Exchange of the *itp1* gene in *U. cynodontis* 2-HP with the *mfsA* gene from *A. terreus* ATEG 09972, G418^R^	this study

Determination of the *cyp3* gene copy number was performed via quantitative real-time PCR. After isolation of genomic DNA, concentrations were adjusted to 0.2 ng μL^−1^. For qPCR, 5 μL of the diluted genomic DNA (corresponds to 1 ng) was mixed with 10 μL 2x Luna® Universal qPCR Master Mix (New England BioLabs, Ipswich, MA, USA) and 1 μL of each oligonucleotide (Table S2) and adjusted to a final volume of 20 μL with ddH_2_O. Measurements were performed in 96-well plates in the qTOWER 2.2 (Analytik Jena, Jena, Germany). For the determination of *cyp3* copy number, the relative concentration of the *cyp3* gene to two single-copy genes (*tad1* and *rdo1*) was calculated via ’Relative quantification method’ of the qPCRsoft 3.1 software (Analytik Jena, Jena, Germany).

## CRediT authorship contribution statement

**Philipp Ernst:** Writing – review & editing, Writing – original draft, Visualization, Validation, Methodology, Investigation, Formal analysis, Data curation, Conceptualization. **Felicia Zlati:** Writing – review & editing, Methodology, Investigation. **Larissa Kever:** Writing – review & editing, Methodology, Investigation, Formal analysis. **Astrid Wirtz:** Writing – review & editing, Methodology, Investigation. **Rainer Goldbaum:** Writing – review & editing, Methodology, Investigation, Formal analysis. **Jörg Pietruszka:** Writing – review & editing, Resources. **Benedikt Wynands:** Writing – review & editing, Supervision. **Julia Frunzke:** Writing – review & editing, Supervision, Resources. **Nick Wierckx:** Writing – review & editing, Supervision, Resources, Project administration, Funding acquisition, Conceptualization.

## Availability of data and materials

All data generated or analyzed during this study are included in this published article and its supplementary information files.

## Funding

This project has received funding from the Bio-based Industries Joint Undertaking (10.13039/501100014757JU) under th e European Union's Horizon 2020 research and innovation program under grant agreement No 887711 for the project Glaukos. The 10.13039/501100014757JU receives support from the European Union's Horizon 2020 research and innovation program and the Bio-based Industries Consortium.

This project was also funded by the 10.13039/501100001659Deutsche Forschungsgemeinschaft (10.13039/501100001659DFG, 10.13039/501100001659German Research Foundation) – Project ID 458090666/CRC1535/1.

## Declaration of competing interest

The authors declare that they have no known competing financial interests or personal relationships that could have appeared to influence the work reported in this paper.

## Data Availability

Data will be made available on request.
