# Supplementary material for: Selective production of the itaconic acid-derived compounds 2-hydroxyparaconic and itatartaric acid
Source: Metab Eng Commun. 2024 Nov 16;19:e00252. doi: 10.1016/j.mec.2024.e00252 (PMC11626831; doi:10.1016/j.mec.2024.e00252)
Supplement: Multimedia component 1 [file mmc1.docx]

# Supplements


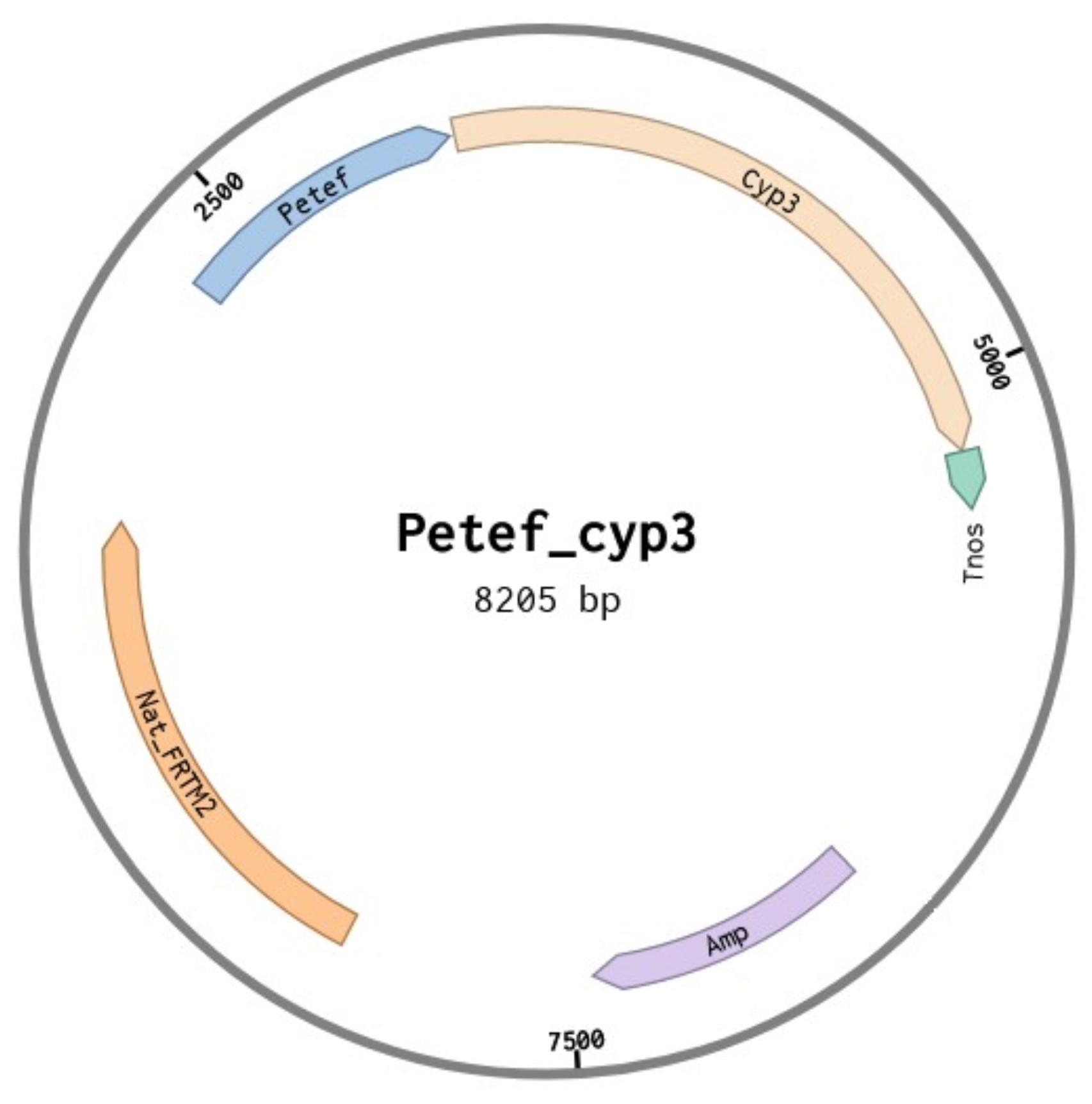


**Figure S1: *P_etef_cyp3* harboring the P450 monooxygenase *cyp3* under the control of the strong, constitutive *P_efef_*promotor.**

A nourseothricin resistance cassette including FRTM2-sites was used. For the random integration into the genome, the plasmid was linearized with FspI.


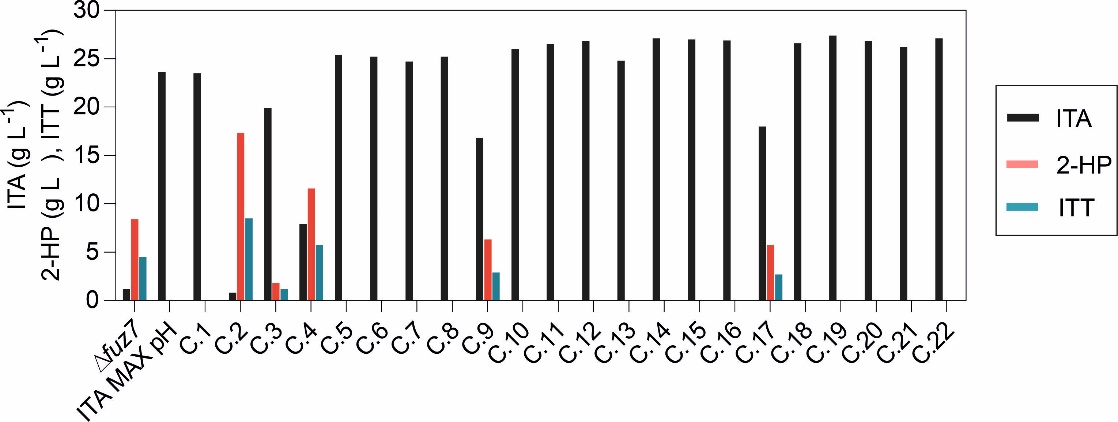


**Figure S2: Initial screening of 22 clones in System Duetz plates – endpoint measurement after 240 h.**

Several clones (22) were cultivated in System Duetz plates in MTM with 15 mM NH_4_Cl, 30 mM MES pH 6.5, and 50 g L^-1^ glucose at 30 °C and 200 rpm (n = 1 biological replicate). ITA (black bar), 2-HP (green bar) and ITT (orange bar).


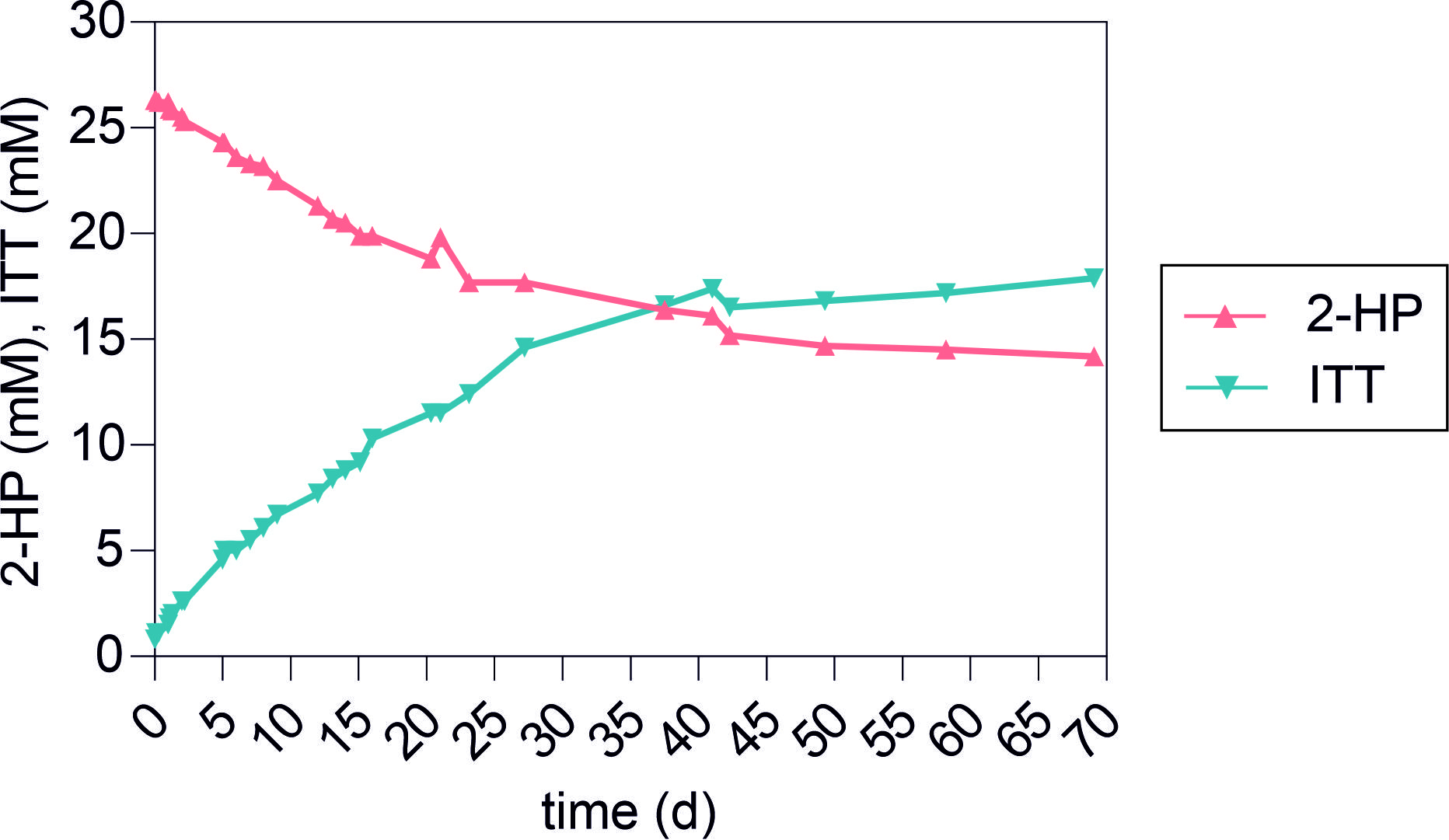


**Figure S3: Non-enzymatic equilibration between 2-HP and ITT under acidic conditions (pH 2.3) at room temperature.** The purified 2‑HP was dissolved in ddH_2_O to approximately 26 mM. No other products were observed by HPLC during this equilibration.


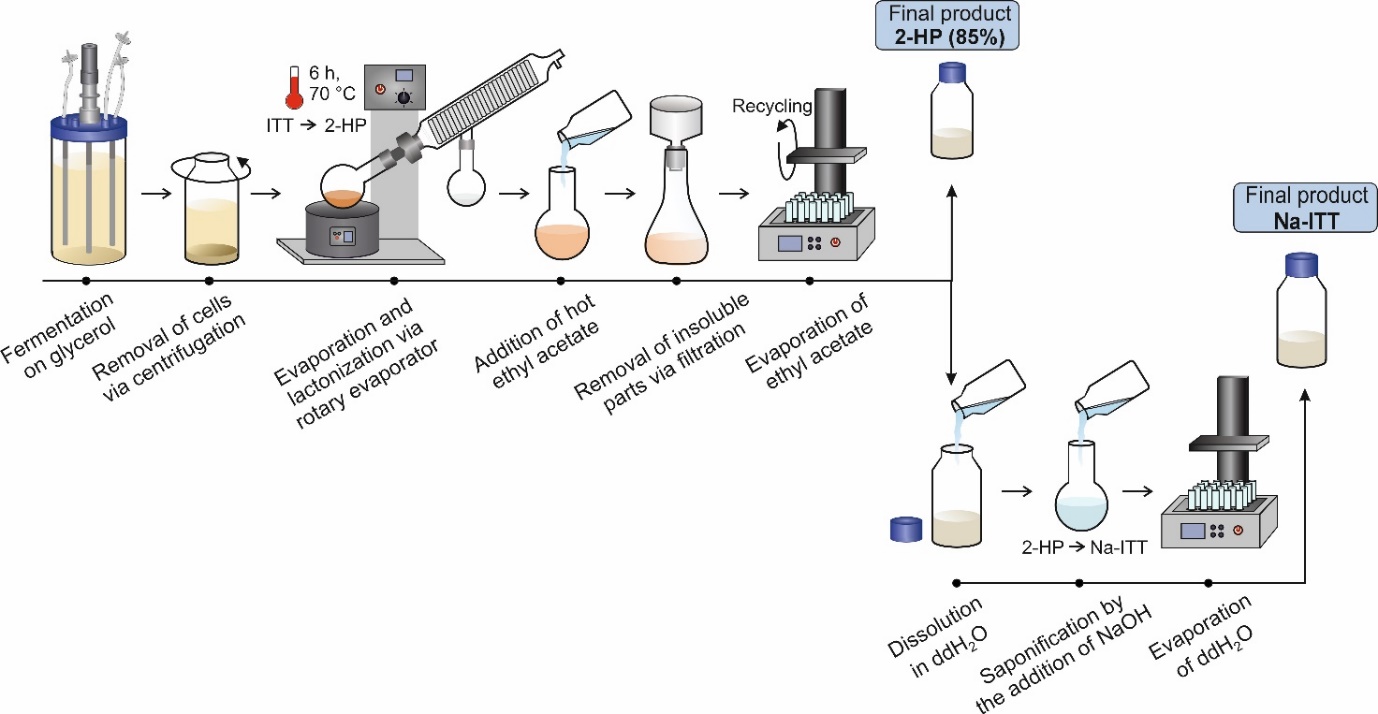


**Figure S4: Schematic representation showing the purification procedure of 2-HP and ITT from culture supernatants.**

2-HP was recovered first through a process of evaporation and lactonization of ITT, followed by 2-HP extraction with ethyl acetate. The obtained 2-HP could subsequently be converted to ITT in the form of its sodium salt by saponification. The shown procedure resulted in 2‑HP with a purity of 85.3 ± 3.7% (rounded to 85% in the scheme) measured via qNMR. The concentration of 2-HP that could be identified in the remaining impurities dissolved in ddH_2_O was lower than 0.5 g L^-1^. In total, 91.7% of the 2-HP initially measured in the fermentation supernatant was recovered. However, when considering the 2-HP produced from ITT lactonization as well, the purification yield was only 49.7%. This indicates that 2‑HP was lost during the purification process, which requires further evaluation in the future.


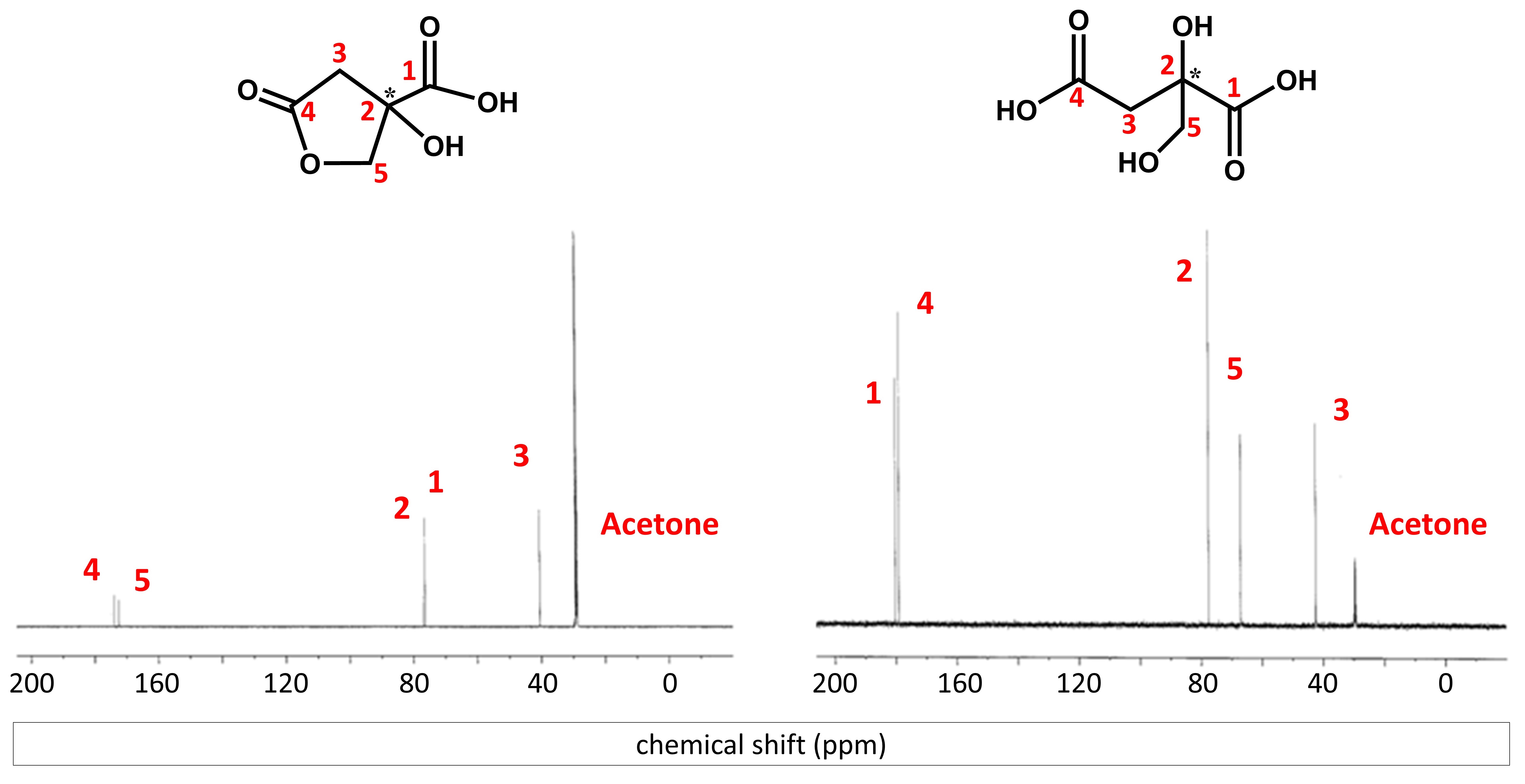


**Figure S5: ^13^C NMR spectra and corresponding structures of 2-HP and ITT. Each peak with different chemical shift and unique shape corresponds to a carbon atom.**

Using ^13^C NMR analysis, the following carbon atoms of 2‑HP were identified: CH_2_ at δ 40.43, CH_2_ at δ 76.47, C(OH)(COOH) at δ 76.84, COOH at δ 172.30 and C=O at δ 173.81. The ^1^H NMR identified the four H atoms of the two CH_2_ groups at the following chemical shifts: 2.63 (^1^H, d), 3.18 (^1^H, d), 4.31 (^1^H, d) and 4.61 (^1^H, d). The ^13^C NMR analysis of ITT identified the following carbon atoms: CH_2_ at δ 42.42, CH_2_ at δ 67.04, C(OH)(COOH) at δ 77.51, COOH at δ 179.08 and COOH at δ 180.27. The ^1^H NMR identified also for ITT the four H atoms of the two CH_2_ groups at the following chemical shifts: 2.39 (^1^H, d), 2.56 (^1^H, d), 3.52 (^1^H, d) and 3.62 (^1^H, d). This is in accordance with the structures published by Guevarra and Tabuchi (1990b). 2-HP was further analyzed by qNMR revealing a purity of 85.30 ± 3.7% (n = 6). The identities of both products 2‑HP and ITT were also confirmed by GC ToF‑MS and DS-FIA-MS/MS.


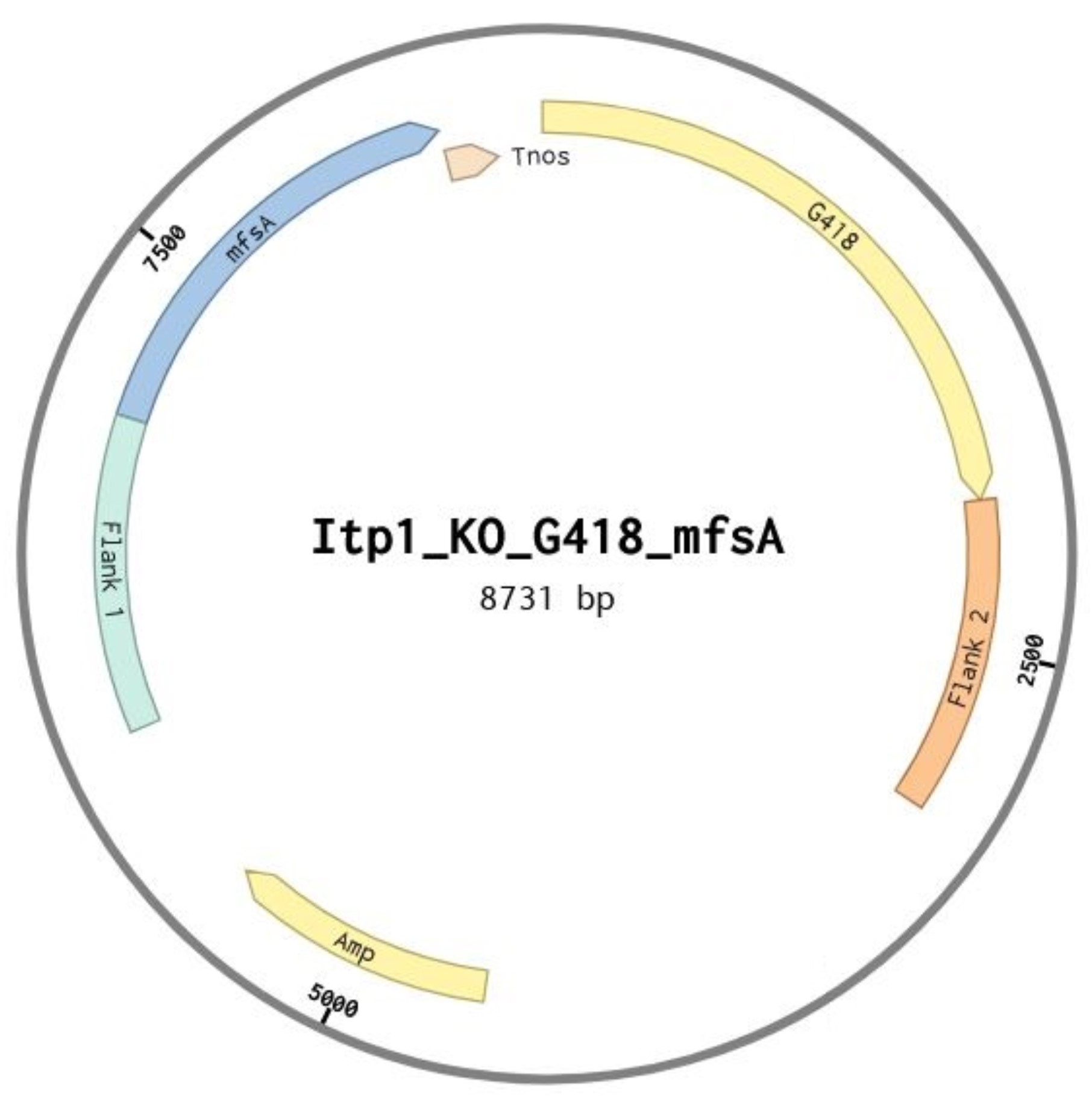


**Figure S6: Construct for the exchange of *itp1* with *mfsA*.**

For the exchange of *itp1* with *mfsA*, homologous recombination with 1000 bp flanking regions and a geneticin G418 resistance cassette were used. The final KO construct was amplified via PCR.


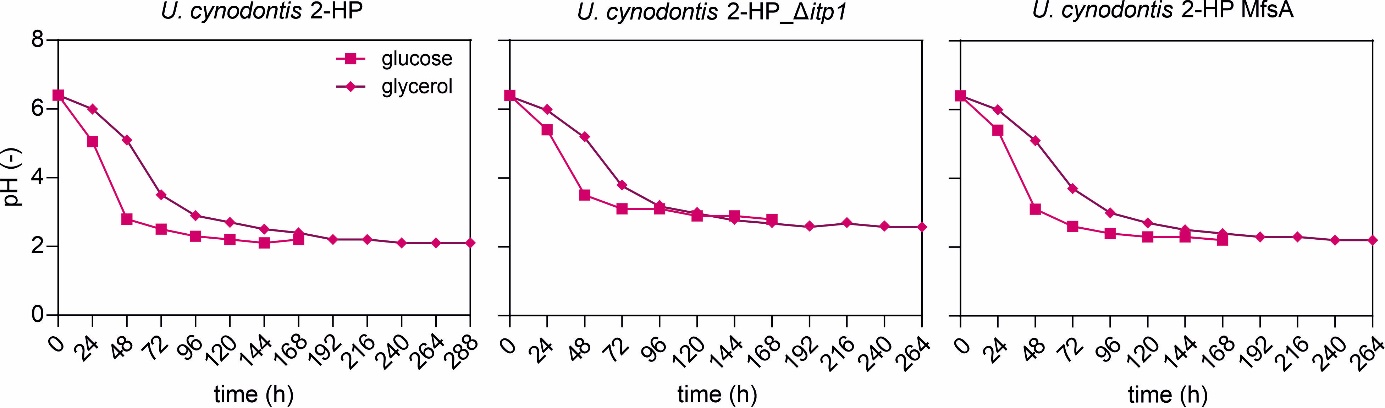


**Figure S7: pH values during System Duetz microcultivations of *U. cynodontis* 2-HP, *U. cynodontis* 2-HP_∆*itp1*, and *U. cynodontis* 2-HP MfsA.** Cultivations were performed in System Duetz plates in MTM medium with 15 mM NH_4_Cl, 30 mM MES pH 6.5, and 50 g L^-1^ glucose or 50 g L^-1^ glycerol at 30 °C and 200 rpm (n = 2 biological duplicates).

**Table S1: Elemental analysis of the purified 2-HP.**

The measured composition correlates well with the expected values. The measured percentages of C, H and O add up to about 99.4 ± 0.3%, meaning that there are likely no other elements in the impurities. Potential contaminants could consist of hydrocarbons, given that the 2-HP samples seem to contain a slight excess of C and H. Moreover, no N was detected, thus excluding protein contaminants from the fermentation. The values indicated for N represent detection limits based on sample weight. In the future, additional purification steps such as activated carbon/celite treatment, nanofiltration, or crystallization may be implemented into the purification process to further increase the purity.

|  | **%C (w w^-1^)** | **%H (w w^-1^)** | **%O (w w^-1^)** | **%N (w w^-1^)** |
| --- | --- | --- | --- | --- |
| 2-HP, expected | 41.1 | 4.1 | 54.8 | 0.0 |
| 2-HP (85.3 ± 3.7%), detected (n=6) | 41.7 ± 0.3 | 4.5 ± 0.1 | 53.2 ± 1.6 | < 0.5 |

**Table S2: Oligonucleotides used for deletion, exchange and overexpression constructs.**

| **Primer name** | **Sequence (5’-3’) and description** |
| --- | --- |
| PE1_fwd | gcctgagtggcctcttgatatatcatatcgttctttcc  Amplification of *cyp3* cassette for the generation of *cyp3* overexpression construct |
| PE2_rev | gaacttctggccggtcggtgtggatgtatg  Amplification of *cyp3* cassette for generation the of *cyp3* overexpression construct |
| PE3_fwd | atccacaccgaccggccagaagttcctattctcaag  Amplification of FRT_M2-NatR cassette for the generation of *cyp3* overexpression construct |
| PE4_rev | tgatatatcaagaggccactcaggccagaag  Amplification of FRT_M2-NatR cassette for the generation of *cyp3* overexpression construct |
| PE5_fwd | ctgagtggcctcctcttcctcgcctctg  Amplification of 5‘-UTR flank for the generation of *itp1* deletion construct |
| PE6_rev | ctagaaagattgccagcagagtcagagag  Amplification of 5‘-UTR flank for the generation of itp1 deletion construct |
| PE7_fwd | tcagcaagatgctggtgccagctctgtatatg  Amplification of 3‘-UTR flank for the generation of *itp1* deletion construct |
| PE8_rev | acttctggccgtcgaggacgttgtttgtg  Amplification of 3‘-UTR flank for the generation of *itp1* deletion construct |
| PE9_fwd | tctgctggcaatctttctagaagatctcctac  Amplification of pJET1.2 backbone for the generation of *itp1* deletion construct |
| PE10_rev | tggcaccagcatcttgctgaaaaactcg  Amplification of pJET1.2 backbone for the generation of *itp1* deletion construct |
| PE11_fwd | cgtcctcgacggccagaagttcctattctctataaag  Amplification of FRT_M7 cassette for the generation of *itp1* deletion construct |
| PE12_rev | aggaagaggaggccactcaggccagaag  Amplification of FRT_M7 cassette for the generation of *itp1* deletion construct |
| PE13_fwd | accatggcgtcgcgaagcttgccggcag  Amplification of G418R cassette for the generation of *itp1* deletion construct |
| PE14_rev | tctgacttgcgccgcactcctacagcttg  Amplification of G418R cassette for the generation of *itp1* deletion construct |
| PE15_fwd | cgtcctcgacatgggtcacggcgacacc  Amplification of *mfsA* cassette for the generation of *itp1*::*mfsA* exchange construct |
| PE16_rev | aagcttcgcgactatagggagaccggcagatc  Amplification of *mfsA* cassette for the generation of *itp1*::*mfsA* exchange construct |
| PE17_fwd | ggagtgcggctcctcttcctcgcctctg  Amplification of backbone for the generation of *itp1*::*mfsA* exchange construct |
| PE18_rev | cgtgacccatgtcgaggacgttgtttgtg  Amplification of backbone for the generation of *itp1*::*mfsA* exchange construct |
| PE19_fwd | tccctatagtcgcgaagcttgccggcag  Amplification of G418R cassette for the generation of *itp1*::*mfsA* exchange construct |
| PE20_rev | aggaagaggagccgcactcctacagcttg  Amplification of G418R cassette for the generation of *itp1*::*mfsA* exchange construct |
| PE21_fwd | acgctaaaagtaaggccgct  Amplification of *cyp3* during qPCR |
| PE22_rev | ggatgggcgatttcctcaca  Amplification of *cyp3* during qPCR |
| PE23_fwd | cgtctacattcacgcttgcg  Amplification of reference gene *tad1* during qPCR |
| PE24_rev | agtgcagccgaagtccaatt  Amplification of reference gene *tad1* during qPCR |
| PE25_fwd | ttgatcttctgcgagccgaa  Amplification of reference gene *rdo1* during qPCR |
| PE26_rev | agcgcgatctatccgtcaag  Amplification of reference gene *rdo1* during qPCR |
